# Lomustine Loaded Superparamagnetic Iron Oxide Nanoparticles Conjugated with Folic Acid for Treatment of Glioblastoma Multiforma (GBM)

**DOI:** 10.22037/IJPR.2020.1101032

**Published:** 2020

**Authors:** Salman Jafari, Mohammad Bagher Tavakoli, Ali Zarrabi

**Affiliations:** a *Department of Radiology, School of Paramedicine, Hamadan University of Medical Sciences, Hamadan, Iran. *; b *Department of Medical Physics, School of Medicine, Isfahan University of Medical Sciences, Isfahan, Iran. *; c *Sabanci University Nanotechnology Research and Application Center (SUNUM), Tuzla 34956 Istanbul, Turkey.*

**Keywords:** Polyglycerol coated SPIONs, Folic acid, Drug delivery, Lomustine, U87- MG cell line

## Abstract

This study aimed to improve delivery of lomustine as a chemotherapeutic agent and to increase its uptake by U87-MG cancer cells via synthesizes LN-FA-PG-SPIONs (lomustine loaded polyglycerol coated superparamagnetic iron oxide nanoparticles conjugated with folic acid). Nanoparticles were synthesized by thermal decomposition method and characterized using TEM (transmission microscope), FTIR (Fourier transform infrared spectroscopy), and VSM (vibrating sample magnetometer). Lomustine release from nanoparticles was determined by dialysis-bag diffusion technique. Nanoparticles cytotoxicity was evaluated by MTT assay. Mean size of SPIONs and FA-PG-SPIONs (PG-SPIONs conjugated with folic acid) were 7.1 ± 1.13 nm and 25.1 ± 3.94 nm, respectively. Based on FTIR spectra SPIONs were successfully coated by polyglycerol and conjugated with folic acid. Lomustine encapsulation efficiency was 46 ± 6.8 %. SPIONs were cytotoxic on U87-MG cells at concentration above 100 ug/ml (*p *< 0.05) but PG-SPIONs do not reduce viability significantly (*p > *0.05). Conjugation of folic acid with PG-SPIONs increased nanoparticles uptake by U87-MG cells (*p < *0.05). We concluded that however FA-PG-SPIONs are proposed as a useful tracer for diagnostic and treatment of GBM but their drug delivery properties for lomustine is not satisfactory and more researches are necessary with this regard.

## Introduction

GBM (glioblastoma multiforma) is known as the most malignant brain cancer with a very poor prognosis (1-3). Standard method for treatment of GBM is currently surgery followed by radiotherapy and concurrent chemotherapy (4). However, there are limitations such as remaining of residual malignant cancer cells in the tumor site after surgery, insufficient dose of chemotherapy agents to the cancer cells, and resistance of tumor cells to treatment (5-7). 

Lomustine is one of the alkylating agents used for treatment of GBM. It is nonspecific for cancer cells and also has effect on rapid dividing normal cells (8, 9). Reducing cytotoxicity and in turn side effects of lomustine requires lowering drug dose but cancer cells should receive enough dose to be killed (10). Targeted drug delivery using nanoparticles has received much attention for treatment of cancers by purpose increasing drug dose to cancer cells while reducing toxicity on normal cells (11-13). 

SPIONs (superparamagnetic iron oxide nanoparticles) have been reported to have good capabilities for drug delivery to cancer cells (14-16). These nanoparticles are appropriate for drug delivery thanks to their targeting property either by an external magnetic field or by targeted molecules as well as drug loading potential (17). 

Folic acid is a targeting agent for which receptors are highly expressed in brain tumors(18, 19). In addition, there are some advantages with it such as high binding affinity to folate receptors, compatibility with variety of organic and aqueous solvents, high stability during storage, low cost and availability (20). Considerable potentials for targeted drug delivery to GBM have been reported for conjugated SPIONs with folic acid (21). 

Coating SPIONs by a suitable material is important for drug delivery applications. Coating SPIONs with a biocompatible polymer can prevent agglomeration of them, minimize protein absorption, and lead to increased circulation time in the blood stream (22-24). HPG (hyperbranched polyglycerol) is a novel polymeric surface coating material that has good chemical stability and excellent biocompatibility (25, 26). It exhibits great promise in the area of biocompatible and bioactive materials for antifouling surfaces and also for controlled drug delivery using nanoparticles. Deng *et al.* suggested that HPG is even superior to PEG, the gold standard coating polymer at present, as a surface coating for drug delivery applications (27).

 Loading lomustine in PG-SPIONs (polyglycerol coated SPIONs) conjugated with folic acid is an interesting idea to resolve insufficient drug dose delivery problem to cancer cells and reduce side effects to normal tissues. In this study we aimed to synthesize LN-FA-PG-SPIONs (lomustine loaded polyglycerol coated superparamagnetic iron oxide nanoparticles conjugated with folic acid) and examine cytotoxicity of these nanoparticles on U87-MG cancer cells.

## Experimental

*Materials*

For synthesize and coating SPIONs by polyglycerol, iron (III) acetylacetonate (Fe (acac) 3) (Merk), ethyl acetate (Merk), triethylene glycol (TREG) (Lobachimi PVT.LTD), and glycidol (MW = 74.08) (ACROS ORGANINCS) were used. For conjugation of folic acid (Fluka) with PG-SPIONs, anhydrous dimethyl sulfoxide (Dry DMSO) (78/13 g/mol), dimethylaminopyridine (DMAP) and dicyclohexylcarbodiimide (DCC) were purchased from Merk. Lomustine (233.70 g/mol) was obtained from Exir-Austria and ethanol from Merk. 

High glucose Dulbecco’s modified eagle’s medium (DMEM) (BioIdia), phosphate buffer saline (PBS) (BioIdia) and penicillin – streptomycin (BioIdia) and fetal bovine serum (FBS) (INCOLON) were used for cell culture. 3-(4, 5-dimethylthiazol-2-yl)-2, 5-diphenyltetrazolium bromide (MTT) (Sigma-Aldrich) and dimethyl sulphoxide (DMSO) molecular grad (Pars Tuse) were used for evaluation of nanoparticles cytotoxicity. U87-MG cell line was obtained from Pasteur institute of Iran. 

*SPIONs synthesis and coating with polyglycerol*

SPIONs were synthesized by thermal decomposition method (25). Briefly 1.06 g of iron (III) acetylacetonate (Fe (acac)3) and 60 mL triethylene glycol (TREG) were mixed in a three necks bottle and magnetically stirred under a slow flow of nitrogen while raising temperature gradually up to 300 °C (28). Next solvent was mixed with 20 mL ethyl acetate and remained on a 0.6 T neoduim magnet for 3 days in order to separate resolved SPIONs. Nanoparticles then freeze dried (VaCo5 ZIRBUS) for use in the next steps (29).

SPIONs were coated with polyglycerol by ring opening reaction of glycidol monomer (25). Thirty milligram SPIONs were mixed with 1 mL of glycidol (MW = 74.08) monomer and sonicated for 30 min. Mixture kept at constant temperature 140 °C under nitrogen gas pressure and magnetically stirred for 20 h. Next 10 mL deionized water was added to the mixture and sonicated for 20 min. PG-SPIONs were separated from solvent by a 0.6 T magnet and dialyzed against deionized water with a dialyze bag (MWCO: 12000 g/mol) and freeze dried for 48 h. 

*Preparation of Conjugated folic acid PG-SPIONs and Loading Lomustine*

In order to prepare FA-PG-SPIONs, briefly 802 mg PG-SPIONs and 100 mg folic acid were dissolved in 5 mL dry DMSO and sonicated for 5 min. Next 30/88 mg DMAP as a catalyst and 59.25 mg DCC as a coupling reagent were added to it. After sonication, beaker was sealed with aluminium foil and the solvent was kept at 5 °C for 36 h under magnetic string. Solvent was then dialyzed against deionized water using a dialysis bag (MWCO: 12000 g/mol) for 24 h and freeze dried for 96 h to obtain FA-PG-SPIONs.

For loading lomustine on FA-PG-SPIONs, briefly 16 mg lomustine and 80 mg FA-PG-SPIONs were dissolved in 2 mL dry DMSO and sonicated for 30 min. Solvent was then dialyzed against a dialysis bag (MWCO: 12000 g/mol) for 24 h. LN-FA-PG-SPIONs (lomustine loaded FA-PG-SPIONs) obtained after freeze drying and were ready for related analysis. 

*Nanoparticles characterizations*

*Size and size distribution*

Transmission microscope (TEM) (Leo 912 AB) images were taken from nanoparticles. Mean nanoparticles size and size distribution of the nanoparticles were determined by MATLAB software (2014a) using TEM images. The results were reported as mean ± SD (standard deviation).

*Fourier transform infrared spectroscopy (FTIR)*

Fourier transform infrared spectroscopy (FTIR) with a resolution of 4 cm^-1^ was performed on nanoparticles by a JACSO 6300 spectrometer. Enough amounts of nanoparticles were mixed with potassium bromide (KBr) to form a tab for analysis. The results were recorded as transmittance spectrum. 

*Vibrating sample magnetometer (VSM)*

Magnetic properties of nanoparticles were measured by vibrating sample magnetometer (VSM) (Meghnatis daghigh kavir kashan). About 100 mg NPs was used for this analysis. 

*UV-Visible spectrophotometry*

This analysis was performed to determine corresponding wavelength of maximum absorbance (λmax), encapsulation efficiency, and examine drug release profile for lomustine. For λmax determination, appropriate amount of lomustine was dissolved in ethanol and scanned by a UV-Visible spectrophotometer (Biowave II) at wavelength 200 to 450 nm. For calculation of encapsulation efficiency 20 mg LN-FA-PG-SPIONs was dissolved in PBS with pH 7.4 and digested with minimum amount of 95% v/v ethanol. After centrifuging at 19400×g (Hettich) for 30 min supernatant was used for analysis. The encapsulation efficiency was calculated using the below Equation:

Encapsulation Efficiency% = 

Time profile for the release of lomustine from the nanoparticles was determined using dialysis bag diffusion technique under magnetic stirring. Forty milligram lomustine loaded nanoparticles were dispersed in 3 mL PBS with pH 7.4 and were dispensed into a dialysis bag with a molecular cut off of 12 kDa. Dialysis bag tied in both sides and suspended in 200 mL PBS at 37 °C with slow continuous magnetic stirring. Three millilitre of release medium was taken at different time intervals and dilated with ethanol. The amount of released lomustine was calculated by measuring light absorbance at 230 nm.

*Biological tests*

*Cell culture*

U87-MG Cells were cultured in T-75 flasks containing high glucose DMEM with 10% fetal bovine serum (FBS) and 1% penicillin/streptomycin and grown in incubator (BINDER) under 5% CO2 pressure at 37 °C temperature. When the cells were well grown and reached to a suitable confluence, they were separated from flask floor by 0.25% trypsin/EDTA solution and were counted for biological tests.

*Atomic absorption spectroscopy (AAS)*

Number of 2 × 10^6^ U87-MG cells were cultured in T-25 flasks and incubated for 24 h. Next SPIONs, PG-SPIONs, and FA-PG-SPIONs contained medium at concentration of 1, 2, and 3 mg/mL were added to the flasks. After 48 h, the cell cultures were carefully washed twice with PBS to remove free nanoparticles. Then, the cells were trypsinized with 0.25% trypsin/EDTA, centrifuged, and the supernatant was removed. Cells pellet resuspended in 1.5 mL a mixture of 37% hydrochloric acid and 69% nitric acid and the final volume increased to 5 mL by deionized water. The centrifuge tubes remained in an ultrasonic bath at 45 °C for at least 4 h. After ultracentrifugation at 19400×g, solution supernatant was used for analysis. The nanoparticles uptake by U87-MG cells was measured by an atomic absorption spectrophotometer (PERKIN-ELMER). AAS results were statistically analysed by Kruskal-wallis test.

*MTT Assay*

Cytotoxicity of nanoparticles on U87-MG cells was examined by MTT test. The cells were cultured in 12-well plates at a concentration of 2 × 10^4^ cells in each well. Five groups were chosen to examine cytotoxicity of nanoparticles based on their concentration including 5, 25, 50,100, and 200 µg/mL and one group was left as control without any nanoparticle. For each concentration three wells were cultured. The nanoparticle containing medium was filtered through a 0.2 µL filter before addition to the wells. The nanoparticle free medium was removed from the wells and the cells were washed twice with PBS and incubated for 24, 48, and 72 h after adding nanoparticles containing medium. After incubation the culture medium was removed and 200 µL fresh medium contained 5 mg/mL 3-(4,5-dimethylthiazol-2-yl)-2,5-diphenyltetrazolium bromide (MTT) was added to each well and remained in incubator for 4 h at 37 °C. Next, the supernatant was carefully removed and 440 µL DMSO was added to each well and remained in darkness for 2 h. The cell viability was examined by measuring light absorbance of the solution using microplate reader (BIO-RAD Model 680) at 570 nm. The cell viability was calculated as percentage of optical density reading of cells treated with nanoparticles divided by the same for untreated cells. Measurements were repeated three times. Kruskal-Wallis test was used for statistical analysis of the MTT test results.

## Results and Discussion

*Nanoparticles characterizations*

**Figure 1:**
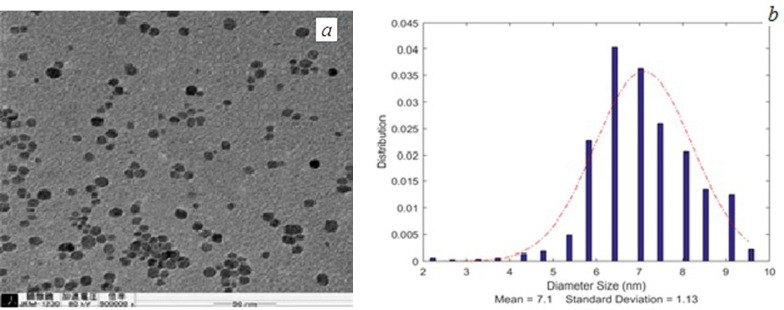
(a) TEM image and (b) size distribution curve of SPIONs

**Figure 2:**
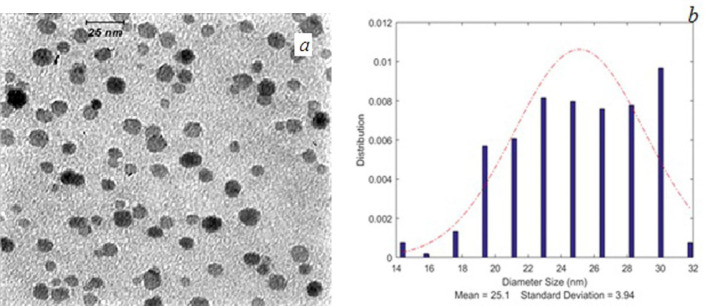
(a) TEM image and (b) size distribution curve of FA-PG-SPIONs

 TEM images of SPIONs and FA-PG-SPIONs are shown in Figures 1 and 2. The mean diameter of SPIONs was 7.1 ± 1.13 nm and it was 25.1 ± 3.94 nm for FA-PG-SPIONs. They were nearly spherical in shape. These ranges of size are suitable for targeted drug delivery applications (30).

For PG-SPIONs which is depicted by red color: Peaks 1100 cm^-1^ and 2900 cm^-1^ are attributed to C-O-C and C-H bonds, respectively, verifying successful coating of SPIONs with glycidol monomer. Peak 900 cm^-1^ is removed on FTIR spectrum for dialyzed PG-SPIONs. This peak is related to epoxy monomers of glycidol which have been eliminated from nanoparticles by dialyze bag. The presence of PG polymer on the surface of SPIONs is verified by peaks around 3300 cm^-1^.

In FA-PG-SPIONs FTIR spectrum (green color) peaks 1607 and 1693 cm^-1^ are related to N-H and C-O bonds, respectively. The presence of phenyl and pterin rings is obvious by peaks in the range 1485-1519 cm^-1^. Those peaks in the range of 3100 to 3500 cm^-1^ are attributed to O-H groups of glutamic acid and N-H groups of pterin ring. These peaks confirmed successful conjugation of PG-SPIONs with folic acid. 

The peaks 1076, 1703, and 3348 cm^-1^ corresponding to N-H, C=O, and N-O-N bonds, respectively, as well as the peaks in the range of 2850-2960 cm^-1^ and 1534-1491 cm^-1^ related, respectively, to CH2=CH2 groups and cyclohexyl ring are obviously observed on LN-FA-PG FTIR spectrum (black color) which confirm presence of lomustine in the nanoparticles. Other peaks for PG-SPIONs and FA-PG-SPIONs are also observed on this spectrum. 

Figure 4 shows PG-SPIONs in a container located in the near of a neoduim magnet. PG-SPIONs have been attracted toward the magnet.

VSM results for investigation of superparamagnetic behaviour and magnetization measurement of SPIONs and PG-SPIONs are shown together in Figure 5. Saturation magnetization for SPIONs and PG coated SPIONs at temperature 25 °C is about 60 and 10 emug^-1^, respectively, which is still suitable for biological applications (31). 

Maximum intensity on UV-Vis absorption spectra of lomustine dissolved in ethanol is located at wavelength 228 nm (Figure 6). Encapsulation efficiency was found to be 46 ± 6.8%. 

LN-FA-PG-SPIONs showed a burst release of lomustine up to 50 h at first but the slope of the curve is then slow. The released lomustine reaches to 67% within 60 h from the start of test. The time to reach fifty percent of maximum (t50%) is about 45 h. Figure 7 shows drug release profile for LN-FA-PG-SPIONs.

Results showed that synthesized nanoparticles have suitable size and size distribution with a superparamagnetic behaviour. Coating causes increase in size and reduction in magnetization of SPIONs which has been already reported in several studies (32-34). This can be due to coating of SPIONs with polyglycerol which reduces SPIONs concentration for analysis and magnetization, consequently (35). Our results were in a good accordance with synthesized SPIONs by Martin *et al*. (34). Presence of similar peaks of 1095 and 2883 cm^-1^ with our study on FTIR spectrum in Wang *et al.* study has been reported to verify successful coating of SPIONs by polyglycerol (36). Dialysis causes removal of excess monomers which did not participate in the reaction. 

Results of FA-PG-SPIONs FTIR spectra verified successful conjugation of folic acid with PG-SPIONs. Presence of similar peaks 1560 and 1652 cm^-1^ in FTIR spectrum of targeted magnetic nanoparticles has been attributed to amid bonds of folic acid in a study by Mohapatra *et al.* (37). In another study by Chen et al peak 1623 cm^-1^ on FTIR spectrum has been claimed to verify successful conjugation of folic acid with PLGA–PEG nanoparticles loaded with vincristine sulfate (38). 

FTIR spectra of LN-FA-PG-SPIONs showed peaks related to those bonds in the structure of lomustine. Encapsulation efficiency of lomustine was relatively low with combination ratio used in our study. Release of lomustine from LN-FA-PG-SPIONs was fast initially but it is followed by a slow slope. Burst release can be related to those molecules which are near the nanoparticles surface. Slow drug release after that is due to hydrophobicity of lomustine which results in retarded drug diffusion (8). 

*Viability tests*

Figures 8A-8E show pictures of the untreated and treated U87-MG cells by nanoparticles which have been taken by an inverted microscope (Nikon Eclipse – TS100).

Results of AAS for evaluation of nanoparticles uptake by U87-MG cells is shown in Figure 9. There is an incense in FA-PG-SPIONs uptake compared with SPIONs and PG-SPIONs at all concentrations (*p < *0.05). Uptake of uncoated SPIONs was the lowest value and did not change with increasing concentration (*p > *0.05). 

Figures 10-12 show viability percentage of U87-MG cells treated with nanoparticles and incubated for 24, 48 and 72 h, respectively. Viability significantly (*p* < 0.001) decreases at concentrations above 100 µg/mL for SPIONs at all times. Increase in PG-SPIONs and FA-PG-SPIONs concentration did not reduce cells viability significantly (*p* > 0.05) as well as PG-SPIONs showed less cytotoxicity compared with other nanoparticles. Significant reduction in cells viability was observed for LN-FA-PG-SPIONs compared with other nanoparticles (*p* < 0.05). For uncoated SPIONs cells viability significantly (*p* < 0.001) decreases at concentrations above 100 µg/mL whose result is also reported in other similar studies (39-41).

Ankamwar *et al.* have reported SPIONs at low concentration have no cytotoxicity effect on U87-MG cancer cells but obvious cytotoxicity is seen at concentration 100 ug/mL (39). Entry of SPIONs into cancer cells leads to formation of reactive oxygen spices (ROS) such as H_2_O_2_, OH^0^ and anion superoxides (O^2-^) which in turn causes oxidative stress and toxicity in cells (10). Viability for U87-MG cells treated with PG-SPIONs is higher compared with other nanoparticles. Viability did not decrease by increasing concentration of PG-SPIONs (*p* > 0.001). In other studies cytotoxicity is not reported even for higher concentration of PG-SPIONs (42). Saatchi *et al.* examined cytotoxicity of PG coated gallium on HUVEC cancer cells and the results showed no significant toxicity effect at concentration of 10 mg/mL (43). Conjugation of folic acid with SPIONs increased uptake of nanoparticles by U87-MG cells. Increase in uptake of SPIONs conjugated with folic acid by MCF-7 cancer cells has been reported by Huang *et al.* which is associated with targeted bonding of folic acid with float receptors (44). LN-FA-PG-SPIONs cytotoxicity was higher than other nanoparticles which is due to presence of lomustine in these nanoparticles.

**Figure 3:**
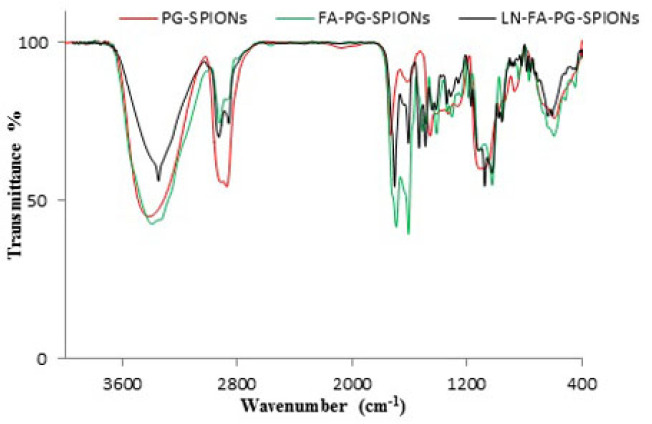
FTIR spectrum of PG-SPIONs (red color), FA-PG-SPIONs (green color) and LN-FA-PG-SPIONs (black color)

**Figure 4:**
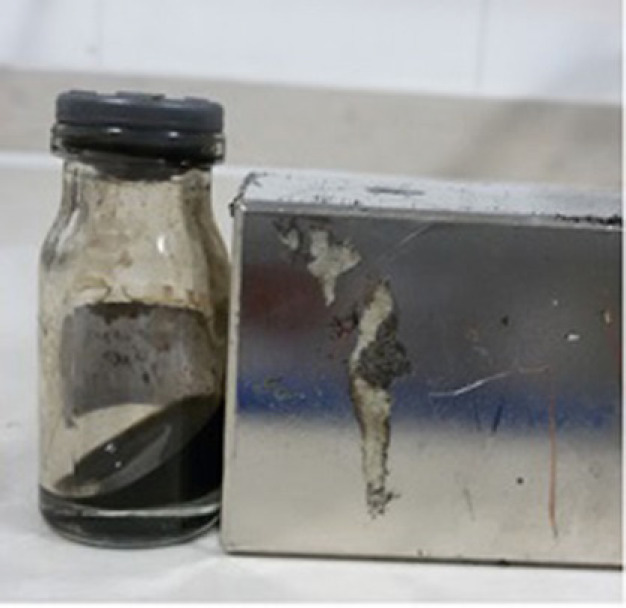
PG-SPIONs attracted by a neoduim magnet

**Figure 5:**
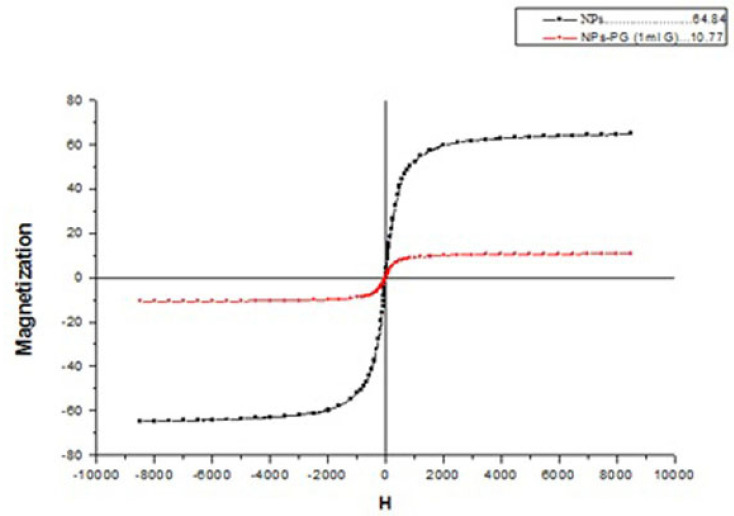
VSM plot for SPIONs and PG-SPIONs

**Figure 6:**
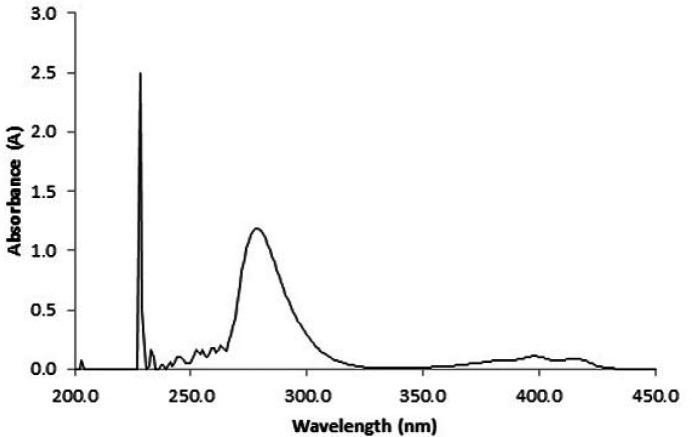
Maximum intensity on UV-Vis absorption spectra of lomustine dissolved in ethanol is located at wavelength 228 nm

**Figure 7:**
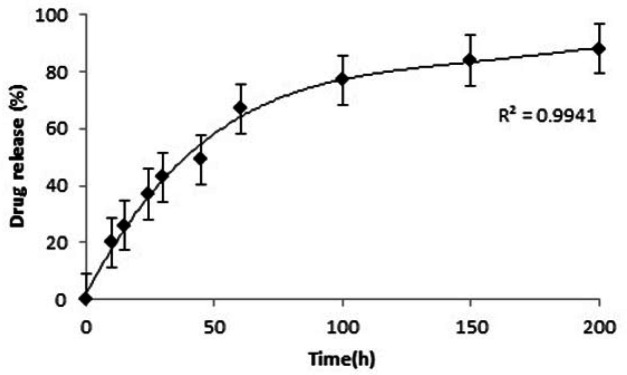
Lomustine release profile for LN-FA-PG-SPIONs

**Figure 8.**
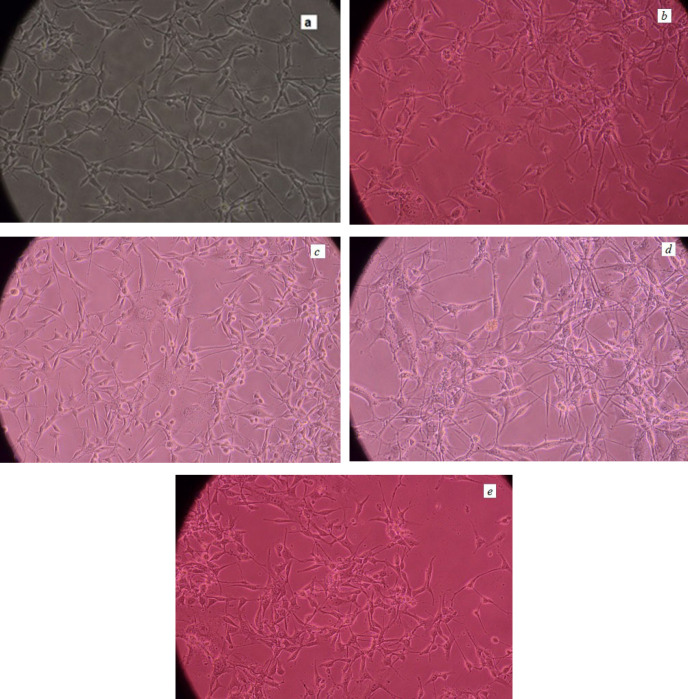
U87-MG cells pictures (a) 48 h post culturing (b) after treating with SPIONs (c) PG-SPIONs (d) FA-PG-SPIONs and (e) LN-FA-PG- SPIONs

**Figure 9:**
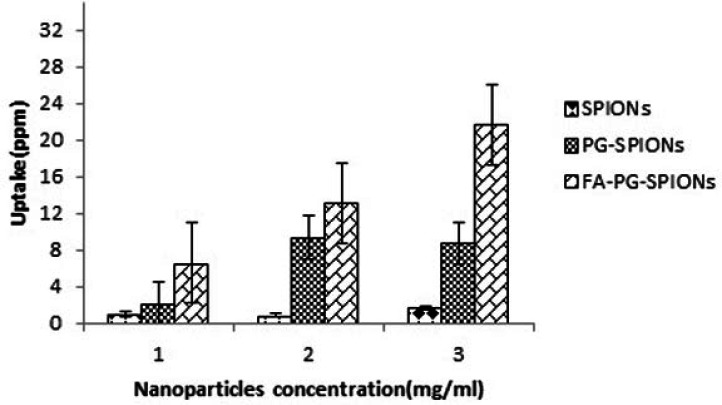
Nanoparticles uptake by U87-MG cells

**Figure 10:**
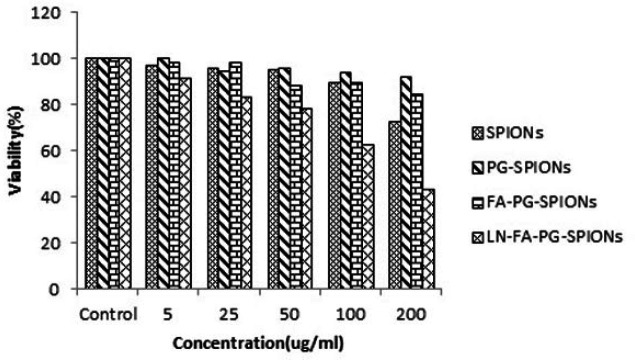
Viability of U87-MG cancer cells treated with nanoparticles for incubation time of 24 h

**Figure 11:**
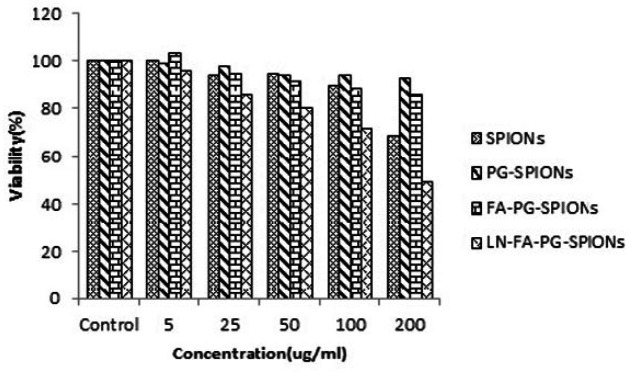
Viability of U87-MG cancer cells treated with nanoparticles for incubation time of 48 h

**Figure 12:**
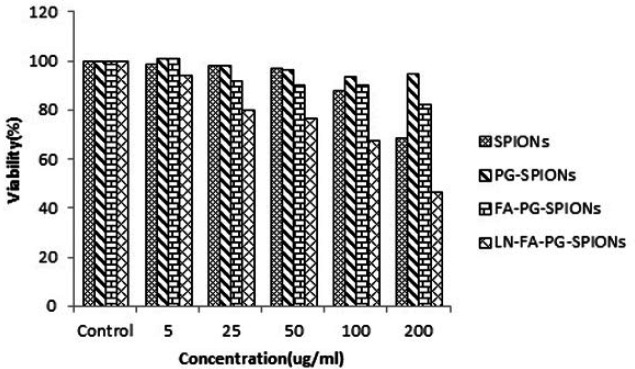
Viability of U87-MG cancer cells treated with nanoparticles for incubation time of 72 h

## Conclusion

This study revealed that SPIONs are cytotoxic on U87-MG cancer cells at concentration above 100 µg/mL but coating them by polyglycerol reduces cytotoxicity. SPIONs conjugation with folic acid increases their uptake by U87-MG cancer cells which provides a promising strategy for diagnostic and treatment of GBM. Lomustine encapsulation efficiency for LN-FA-PG-SPIONs is relatively low with ratio used in our study but more studies should be conducted in this regard. Results of MTT test showed that cell viability decreases significantly by loading lomustine in FA-PG-SPIONs. Based on our results we conclude that however FA-PG-SPIONs are proposed as a useful tracer for cancer diagnostic and treatment of GBM but their drug delivery properties for lomustine are not satisfactory and more researches are necessary to be conducted in this regard.
